# Dependency of B-1 Cells in the Maintenance of Splenic Interleukin-10 Producing Cells and Impairment of Macrophage Resistance in Visceral Leishmaniasis

**DOI:** 10.3389/fmicb.2017.00978

**Published:** 2017-06-02

**Authors:** Angélica Fernandes Arcanjo, Dirlei Nico, Gabriellen Menezes Migliani de Castro, Yasmin da Silva Fontes, Paula Saltarelli, Debora Decote-Ricardo, Marise P. Nunes, Antônio Ferreira-Pereira, Clarisa B. Palatnik-de-Sousa, Célio G. Freire-de-Lima, Alexandre Morrot

**Affiliations:** ^1^Instituto de Biofísica Carlos Chagas Filho, Universidade Federal do Rio de JaneiroRio de Janeiro, Brazil; ^2^Departamento de Microbiologia Geral, Universidade Federal do Rio de JaneiroRio de Janeiro, Brazil; ^3^Instituto de Veterinária, Universidade Federal Rural do Rio de JaneiroRio de Janeiro, Brazil; ^4^Instituto Oswaldo Cruz, FiocruzRio de Janeiro, Brazil; ^5^Departamento de Imunologia, Instituto de Microbiologia, Universidade Federal do Rio de JaneiroRio de Janeiro, Brazil

**Keywords:** visceral leishmaniasis, *Leishmania (L.) infantum chagasi*, B-1 cells, host protective responses

## Abstract

Visceral leishmaniasis is a neglected disease caused by *Leishmania* protozoa parasites transmitted by infected sand fly vectors. This disease represents the second in mortality among tropical infections and is associated to a profound immunosuppression state of the host. The hallmark of this infection-induced host immunodeviation is the characteristic high levels of the regulatory interleukin-10 (IL-10) cytokine. In the present study, we investigated the role of B-1 cells in the maintenance of splenic IL-10 levels that could interfere with resistance to parasite infection. Using an experimental murine infection model with *Leishmania (L.) infantum chagasi* we demonstrated an improved resistance of B-1 deficient BALB/XID mice to infection. BALB/XID mice developed a reduced splenomegaly with diminished splenic parasite burden and lower levels of IL-10 secretion of purified splenocytes at 30 days post-infection, as compared to BALB/c wild-type control mice. Interestingly, we found that resident peritoneal macrophages isolated from BALB/XID mice were more effective to control the parasite load in comparison to cells isolated from BALB/c wild-type mice. Our findings point to a role of B-1 cells in the host susceptibility to visceral leishmaniasis.

## Introduction

Visceral leishmaniasis (VL), also known as Kala Azar is a neglected tropical disease caused by the intracellular protozoan *Leishmania donovani* and *Leishmania (L.) infantum chagasi* parasites (Kaye and Scott, 2011). Over 90% of the annual incidence of new cases occurs in Bangladesh, India, Nepal, Sudan, South Sudan, Ethiopia, and Brazil. In these countries, the outbreaks and prevalence of infection, from which are reported clinical cases, differ in their eco-epidemiology and sand fly vectors involved. This disease is fatal if not treated, and can kill between 20,000 and 40,000 people a year worldwide. The treatment is often performed on the basis of pentavalent antimony compounds and amphotericin B lipid formulations, and its symptoms include: hepatosplenomegaly, fever, anemia, weight loss, and hyperglobulinemia (Kaye and Scott, 2011; Matlashewski et al., 2011; McCall et al., 2013; Ready, 2014).

The immune system works as a crucial barrier in the hosts to the establishment of natural infections. The initial steps of a immune response against *Leishmania* infection is triggered from the activation of innate receptors pattern recognition receptors (PRRs) by molecules associated with pathogens (MMAPs) such as lipophosphoglycans, glycoinositolphospholipid, and metalloproteinase GP63, all expressed on parasite cell surface (Liu and Uzonna, 2012). Activation of PRRs is crucial for induction of interleukin-12 (IL-12) by antigen presenting cells necessary to promote the secretion of interferon-gamma (IFN-γ) by CD4^+^ T lymphocytes and natural killer cells. IFN-γ is a type-1 pro-inflammatory cytokine extremely important to activate the microbicidal activity of macrophages, the major reservoir of *Leishmania* parasites. Once activated, macrophages are able to secrete reactive oxygen species (ROS) and nitric oxide (NO), both involved in the destruction of parasites (Kaye and Scott, 2011; Liu and Uzonna, 2012).

In natural and experimental VL infection, cell-mediated immune responses are suppressed causing a decrease in IFN-γ levels. This subversion of the immune response is associated with production of regulatory cytokines such as interleukin-10 (IL-10) and transforming growth factor-beta (TGF-β), associated with the progression of disease (Kumar and Nylén, 2012). In human infection, significantly higher levels of IL-10 produced by regulatory T cells are present in patients that do not respond to chemotherapeutic treatment, suggesting an important role of this cytokine in the suppression of host immunity during disease (Guha et al., 2014). Increased levels of IL-10 negatively modulate innate immunity via macrophage inhibition of ROS and NO expression (Kumar and Nylén, 2012).

The expression of IL-10 is not specific to cells of the innate immune system but also lymphocytes, including B cells that mediate suppressive responses in VL (Murphy et al., 2001; Deak et al., 2010; Gautam et al., 2011; Bankoti et al., 2012). It has been shown that IL-10-derived from B cells is capable to promote the development of suppressive responses associated with susceptibility to infection (Bankoti et al., 2012; Arcanjo et al., 2015). However, the identification of the B cell population involved in the susceptibility to VL is still vague and needs further studies. Recently it has been demonstrated that B-1 cells contribute to susceptibility to infection with *L. (L.) infantum chagasi* (Gonzaga et al., 2015). B-1 cells represent the major population of B lymphocytes in the pleural and peritoneal cavity. These cells are able to secrete high levels of IL-10 that could modulate the phagocytic activity of macrophages (Aziz et al., 2015). The impairment of the mononuclear phagocyte system is a key factor in the disease progression thus contributing to splenic dysfunction and symptoms of splenomegaly (Kaye et al., 2004). In the present study, we aimed to investigate the role of B-1 cells in the resistance of macrophages to *Leishmania* infection.

## Materials and Methods

### Ethics Statement

All mouse studies followed the guidelines set by the National Institutes of Health, United States. The study was approved by the Research Ethics Committee of Federal University of Rio de Janeiro (protocol IMPPG040-07/16). Protocols for animal were approved by the Institutional Ethical Committees in accordance with international guidelines. All animal experimentation was performed in accordance with the terms of the Brazilian guidelines for the animal welfare regulations.

### Animals, Infection, and Evaluation of Host Responses

BALB/c wild-type control mice and BALB/XID mice (X-linked BALB/c immunodeficient mice genetically deficient in B-1 cells) originated from breeding colonies kindly donated by Professor Mário Mariano (UNIFESP, Brazil) were maintained in our animal facilities (UFRJ). Experimental infection was performed by inoculating 4- to 8-week-old female BALB/c and B-1 cell-deficient BALB/XID mice intravenously with 5 × 10^7^ *L. (L.) infantum chagasi* amastigotes (IOC-L 3324) obtained from infected hamster spleens. Thirty days after infection, mice were euthanized and the liver and splenic parasite load were evaluated in Giemsa-stained smears and expressed in LDU values (*Leishman–Donovan* units of Stauber = number of amastigotes per 1000 liver cell nuclei/mg of liver weight).

### Anti-*Leishmania (L.) infantum chagasi* ELISA

Immunoglobulin isotypes and IgG subtype profiles were monitored by an enzyme-linked immunosorbent assay (ELISA) using the freeze and thawed lysate of stationary phase promastigotes of *L. (L.) infantum chagasi* (MHOM/BR/74/PP/75) as antigen. Whole parasite antigens (2 μg/ml) were plated at 100 μl/well to 96-well plates and, after overnight incubation at 4°C, the plates were washed three times using PBS containing 0.05% (vol/vol) Tween 20 (Sigma, Gillingham, United Kingdom). Serial twofold 1:100 to 1:800 dilutions of serum samples from mice diluted in PBS containing 0.05% Tween were added to the plates and incubated at 37°C for 1 h. Afterward the plates were washed three times with PBS containing 0.05% Tween, and the 1:5,000 dilution of peroxidase-labeled each goat anti-mouse Ig isotypes (Jackson ImmunoResearch, West Grove, United States) were added at 100 μl/well and incubated at 37°C for 1 h. The reaction was developed with 50 mM phosphate/citrate buffer (pH 5.0) containing 2 mM *o*-phenylenediamine HCl and 0.007% (vol/vol) H_2_O_2_ (Sigma, United Kingdom), and interrupted with the addition of 2 M H_2_SO_4_ (50 μl/well). The ELISA plates were read at 490 nm (Spectra Max 190, Molecular Devices, Sunnyvale, United States).

### Cytokine Assays

Splenocytes (1 × 10^6^/0.5 mL) obtained from control or infected mice at 30 days post-infection (DPI) were cultured in 48 well at 37°C/5% CO_2_ in complete RPMI medium, stimulated or not with 10^6^ freeze–thawed stationary phase *L. (L) infantum chagasi* (L579 Fiocruz) promastigotes. After 3 days, supernatants were collected and cytokine levels (IFN-γ, TNF-α, IL-10, and TGF-β) were assayed by ELISA (R&D Systems). Plates were read at 405 nm and values are presented as pg cytokine/mL [mean ± standard error (SE)]. Statistical differences between mean values were evaluated by ANOVA, and pair-wise comparisons were done by the Tukey test.

### *In Vitro* Infection of Macrophages and Detection of ROS

Resident peritoneal macrophages isolated from BALB/c or BALB/XID mice cultured at 1.0 × 10^5^ cells/well in 48-well plates received 10^6^ *L. (L.) infantum chagasi* promastigote forms (MHOM/BR/74/PP/75) in the stationary phase at 37°C in complete Dulbecco’s Modified Eagle’s Medium (DMEM) containing 10% fetal bovine serum (FBS). After 4 h, the cell monolayers were extensively washed for the removal of extracellular parasites, and the cultures were then maintained for 3 days at 37°C. Afterward, infected macrophages were cultured in Schneider medium (Life Technologies) supplemented with 20% FBS at 26°C for an additional 3 days in order to estimate the *L. chagasi* load by counting the promastigotes forms, as described elsewhere (Ribeiro-Gomes et al., 2004). For detection of ROS, cells were incubated with 10 μM H_2_DCFDA probe (Invitrogen) prior to parasite infection and/or activation stimuli (200 ng/ml LPS, 2 ng/ml IFN-γ). A change in fluorescence was assessed with a fluorimeter (Spectramax M3).

### Statistical Analysis

Statistical analyses were performed with GraphPad Prism 4 software, using one-way ANOVA test. Results were expressed as mean ± SE, differences between control and treated group were considered statistically significant when *p* ≤ 0.05.

## Results and Discussion

**FIGURE 1:**
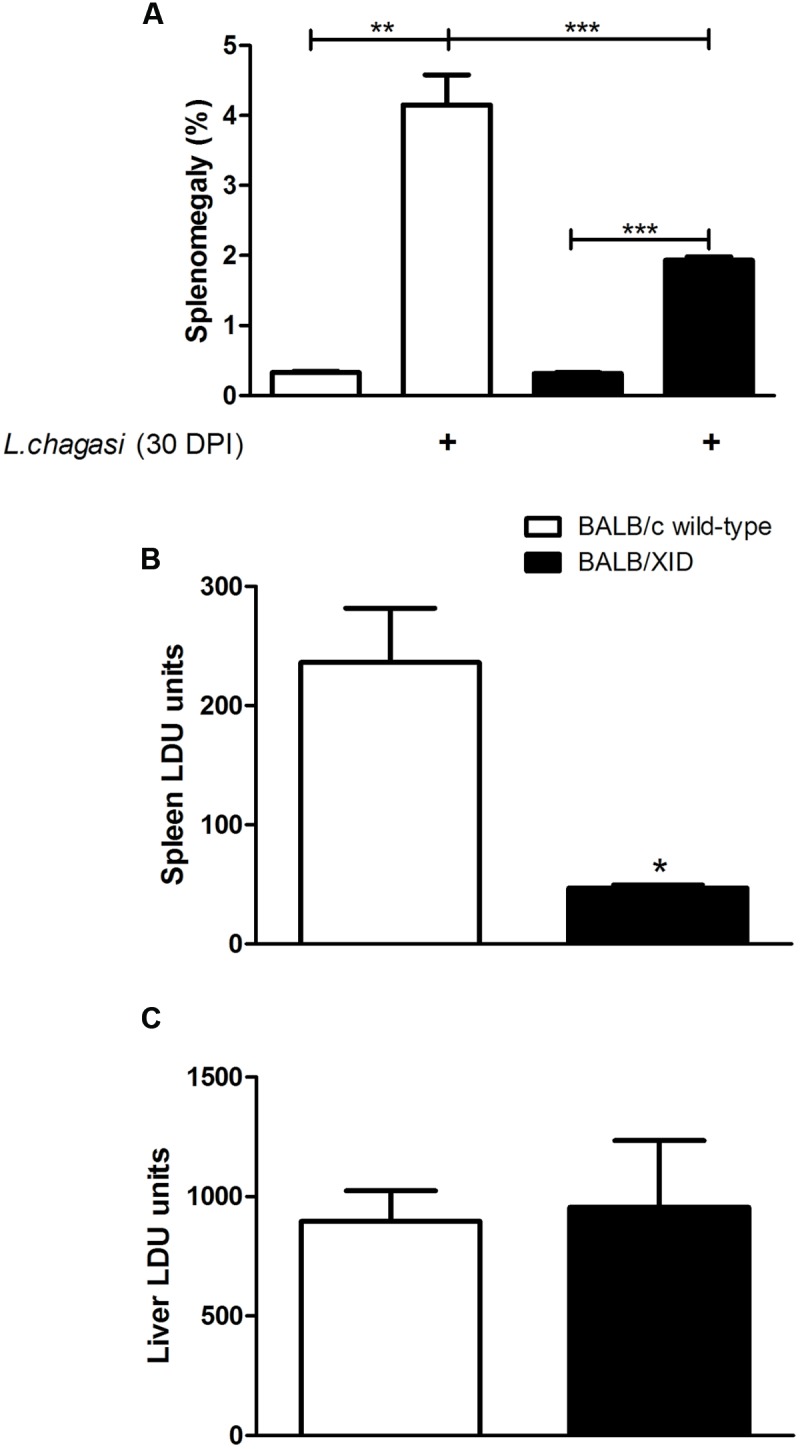
B-1 cell-deficient BALB/XID mice show resistance to visceral leishmaniasis. BALB/c and BALB/XID mice were intravenously injected with 5 × 10^7^ amastigotes of *Leishmania (L.) infantum chagasi*, and 30 days after infection the clinical signs of disease were examined. **(A)** Spleen/body relative weights are attenuated in B-1 cell-deficient BALB/XID mice. The body weight was measured in grams and the spleen/body relative weights (grams of organ weight × 100/grams of body weight) were determined in BALB/c and BALB/XID mice. **(B)** Decreased parasite burden in the spleen of BALB/XID mice. The vertical axis in the histogram represents the average parasite load from spleen or **(C)** liver tissues in Leishman–Donovan units of Stauber (LDU = number of amastigotes/1000 cell nuclei × organ weight in mg) obtained at 30 days post-infection (DPI). Data are means ± SE and represent the results of two independent experiments performed with five to six mice per group. Differences between groups are significant ^∗^*p* < 0.05, ^∗∗^*p* < 0.01, ^∗∗∗^*p* < 0.001.

**FIGURE 2:**
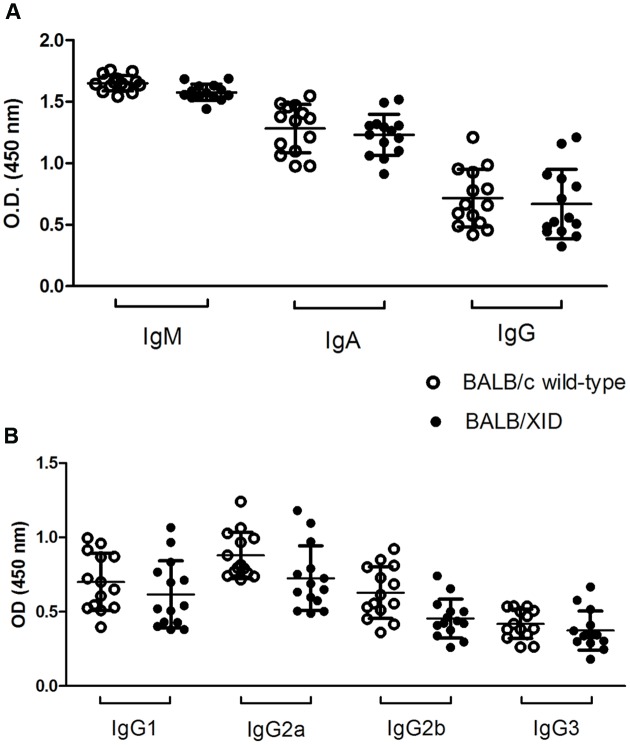
Isotype and IgG subclass profiles of serum antibodies to *Leishmania (L.) infantum chagasi* of B-1 cell-deficient mice. Sera from wild type BALB/c and B-1 cell-deficient BALB/XID mice infected with 5 × 10^7^ amastigote forms of *Leishmania (L.)* infantum chagasi were collected at 30 DPI and the absorbance values of **(A)** antibody Isotypes (IgM, IgA and IgG); and **(B)** IgG antibody subclasses (IgG1, IgG2a, IgG2b and IgG3) were determined by ELISA using *Leishmania (L.) infantum chagasi* promastigote lysates as parasite antigens. Bars show levels of immunoglobulin isotypes and IgG subclasses as the individual absorbancy values of 1/100 diluted sera. Data represent the individual results for each group of mice obtained from two independent experiments.

In our study, we used a murine model of VL in which infection of BALB/c mice with *L. (L.) infantum chagasi* amastigotes gives rise to a higher parasite load in the first weeks of infection, after which it is controlled by the host immune response. The infection of BALB/c and B-1 cell-deficient BALB/XID mice was performed by inoculating 4- to 8-week-old females intravenously with 5 × 10^7^ *L. (L.) infantum chagasi* amastigotes (IOC-L 3324) obtained from infected hamster spleens. Thirty days after infection, mice were euthanized and the splenic and intrahepatic parasite burdens were evaluated in Giemsa-stained smears and expressed in LDU values. Our results demonstrated an increased resistance of BALB/XID mice correlating with lower increases in the spleen/body weight ratio as compared to BALB/c wild-type control mice (**Figure 1A**). These results were in line with the demonstration of a reduced splenic parasite burden at 30 DPI in B-1 cell-deficient mice (**Figure 1B**). In contrast, infection in both mice groups yielded similar intrahepatic parasite burden indexes 1 month after intravenously inoculation with *L. (L.) infantum chagasi* amastigotes (**Figure 1C**), corroborating previous findings using an infection experimental model with lower parasite dose (Gonzaga et al., 2015). The higher susceptibility of infected BALB/XID mice was not due to any alteration of antibody-mediated responses as we did not observe any significant change in the immunoglobulin isotypes nor in the IgG subtype profiles from both groups at 30 DPI (**Figure 2**).

**FIGURE 3:**
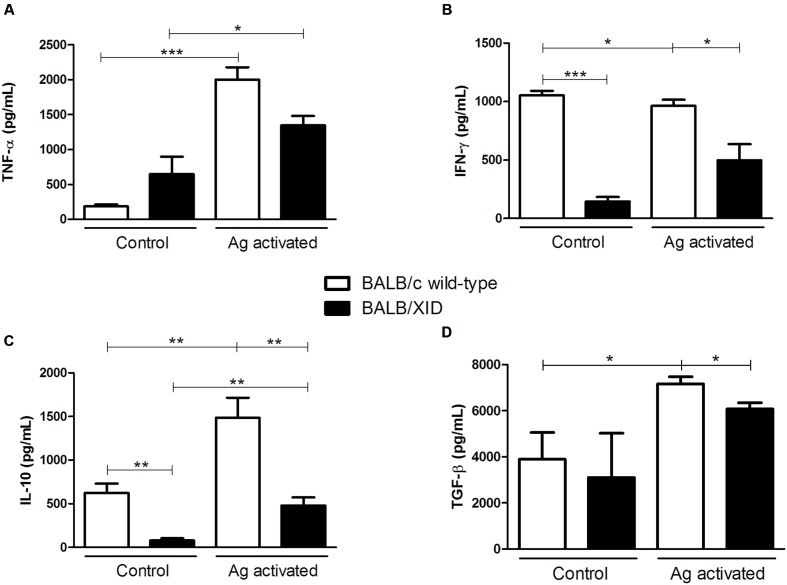
Splenocytes from B-1 cell-deficient BALB/XID mice produce low levels of IL-10. BALB/c and BALB/XID mice were intravenously infected with 5 × 10^7^ amastigotes of *Leishmania (L.) infantum chagasi*, and 30 days after infection splenocytes were isolated and assayed for cytokine production upon parasite antigen (Ag) stimulation. After 3 days of *in vitro* culture with freeze and thawed lysates of *Leishmania (L.) infantum chagasi* promastigotes, as described elsewhere (Popi et al., 2012), splenocyte supernatants were harvested for determination of **(A)** TNF-α, **(B)** IFN-γ, **(C)** IL-10, and **(D)** TGF-β by ELISA. The *y*-axis represents the levels of cytokines, detected by specific ELISA assays, expressed in ng/ml. Data are means ± SE and represent the results of two independent experiments performed with five to six mice per group. Asterisks represent statistical significance between groups ^∗^*p* < 0.05, ^∗∗^*p* < 0.01, ^∗∗∗^*p* < 0.001.

A major factor contributing to susceptibility in leishmaniasis is the development of a strong IL-10 response (Murphy et al., 2001; Gautam et al., 2011; Bankoti et al., 2012). We therefore compared the cytokine responses to infection with *L. (L.) infantum chagasi* parasites by analyzing the supernatants of splenocytes isolated from both infected BALB/XID mice and BALB/c wild-type control groups that were stimulated with whole parasite antigens for 3 days. Splenocytes obtained from both infected mice groups at 30 DPI showed increased levels of protective pro-inflammatory response characterized by high levels of TNF-α, when antigen-stimulated (**Figure 3A**). However when compared the ratio of antigen-stimulated/control indexes for the TNF-α values, we observed a higher increase for BALB/c wild-type control groups as compared to BALB/XID mice. Analysis of IFN-γ expression also indicated an increased levels of this pro-inflammatory cytokine upon stimulation of splenocytes with parasite antigens in BALB/XID mice, although basal levels of this cytokine were secreted in the controls of BALB/c wild-type mice (**Figure 3B**). Increased basal levels of IFN-γ may indicate a progressive disease with high parasite load and infection-induced pathology (Goto and Prianti, 2009). In contrast, the splenocytes from infected BALB/XID mice produced lower levels of IL-10 upon stimulation with parasite antigens (**Figure 3C**). The same pattern was obtained for the regulatory TGF-β cytokine, as the splenocytes from infected BALB/XID mice produced slightly lower amounts of TGF-β upon stimulation with parasite antigens in comparison to controls of BALB/c wild-type mice (**Figure 3D**). However, although statistically significant, the differences observed for TGF-β between both mice groups have not yielded striking results.

**FIGURE 4:**
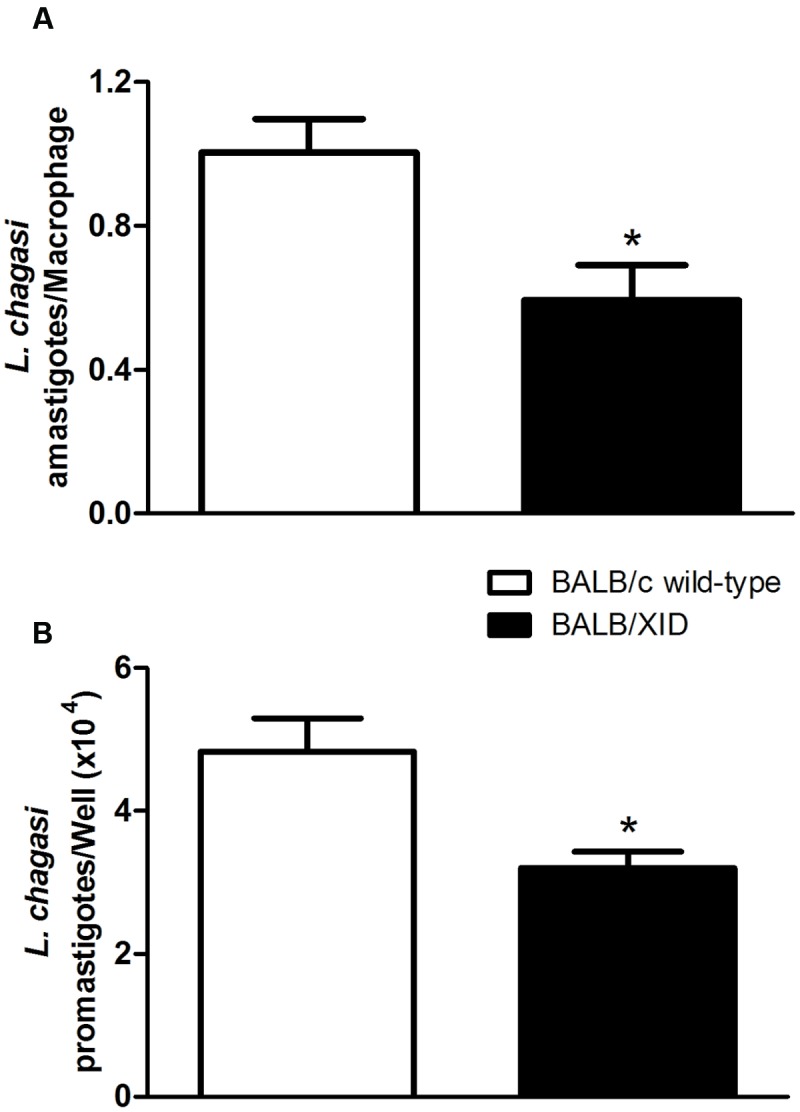
*Leishmania (L.) infantum chagasi* infection of tissue-resident macrophages obtained from BALB/c and BALB/XID mice. Intraperitoneal macrophage cells purified from BALB/c and BALB/XID mice were cultured at 1.0 × 10^5^ cells/well in 48-well plates and infected with 10^6^ *Leishmania (L.) infantum chagasi* promastigotes at a 10:1 ratio of parasites:host cells in DMEM containing 10% FBS at 37°C. The infected cells were washed 4 h to remove extracellular parasites and then maintained for 3 days at 37°C to determine the intracellular amastigote load **(A)**. Following intracellular parasitism, infected macrophages were cultured in Schneider medium supplemented with 20% FBS at 26°C for an additional 3 days to estimate the *L. chagasi* load by counting the promastigotes forms derived from released parasites **(B)**. Histograms represent the means ± SE of total number of *Leishmania (L.) infantum chagasi* forms of triplicate assays and are representative from two independent experiments. Differences between groups are significant ^∗^*p* < 0.05.

We next tested the capacity of resident peritoneal macrophages isolated from BALB/XID mice to control de parasite load in comparison to their counterparts isolated from infected BALB/c wild-type mice. In these assays, macrophages were *in vitro* infected with *L. (L.) infantum chagasi* promastigote forms and the parasite load was measured to determine the macrophage resistance to *Leishmania* infection. According to our results, resident peritoneal macrophages obtained from BALB/XID mice were more effective in eliminating *Leishmania* parasites as compared to macrophages isolated from BALB/c wild-type mice (**Figure 4**). The differences in the innate resistance from resident peritoneal macrophages isolated from both mice groups were not due to any chance in the production of ROS, which are important effector agents against intracellular pathogens. Our results showed that *in vitro*-infected IFN-γ/LPS-activated macrophages from both mice groups produced ROS within first hours of infection with *L. (L.) infantum chagasi* promastigotes (**Supplementary Figure S1**). The data point to the interference of inhibitory effect of homeostatic IL-10 produced by B1-cells on the resident peritoneal macrophages derived from BALB/XID mice.

Taken together, our results clearly indicate that BALB/c mice have an impaired immune response upon infection with *L. (L.) infantum chagasi* as compared to BALB/XID mice. The maintenance of low levels of IL-10 in BALB/XID mice due to the loss of B-1 cells is associated with an improved control of the parasite in spleen tissues and enhanced innate resistance of macrophages to *Leishmania* infection. These results indicate a potentiation of the anti-parasitic activity in B-1 cell-deficient BALB/XID mice. The role of B-1 cells in protective mediated-immunity of the host depends on the nature of the pathogen as well as the infection experimental models (Minoprio et al., 1993; Hoerauf et al., 1994; Babai et al., 1999; Gaubert et al., 1999; Herbert et al., 2002; Popi et al., 2008; Crane et al., 2013; Szymczak et al., 2013; Gonzaga et al., 2015). Intracelular parasites such as *Trypanosoma cruzi* and *Francisella tularensis* that target macrophage cells are susceptible to negative modulation of the mononuclear phagocyte system by B-1 cells (Minoprio et al., 1993; Crane et al., 2013). In fact these cells can be programmed to differentiate into phagocytes (Popi et al., 2012) that could promote immunosurveillance in the different tissues affected by the parasitism thus contributing to the outcome of infection.

Alternatively, B-1 cells can differentiate into IgM-secreting cells working as an innate-like B cell populations with distinct repertoire and tissue location from the conventional B lymphocytes or B-2 cells (Aziz et al., 2015). Interestingly, it has been proposed that the production and activation of a polyclonal B-lymphocyte responses expressing IgM are the major cause of disease susceptibility in animals infected with *L. infantum* (Deak et al., 2010). In addition, it has been shown that B cells in the marginal zone are able to suppress antigen-specific responses from both CD8^+^ and CD4^+^ lymphocytes during the early stages of VL, thereby preventing the generation of protective effector and memory T cells in *L. donovani* infection (Bankoti et al., 2012). The properties of B-1 cells to act as rapid immune responders that promptly migrate and redistribute to secondary lymphoid tissues where they can be resident as cytokine and antibody-secreting differentiated cells (Aziz et al., 2015) could be determinant to the development of infection. Understanding the role of B-1 cells and the control of infection-induced signals that activate these cells in VL could lead to better strategies to control this devastating disease.

## Author Contributions

AM, CF conceived and designed the experiments. AA, DN, GC, YS, PS performed the experiments. AA, DN, PS, DD, MN, CF and AM analyzed the data. DD, AF, CP and CF contributed reagents/materials/analysis tools. CF and AM wrote the paper.

## Conflict of Interest Statement

The authors declare that the research was conducted in the absence of any commercial or financial relationships that could be construed as a potential conflict of interest.

## Supplementary Material

FIGURE S1 ROS production during *Leishmania (L.) infantum chagasi* promastigote infection. Resident peritoneal macrophages isolated from BALB/c or BALB/XID mice were infected with *Leishmania (L.) infantum chagasi* promastigote forms (at 1:10 host–parasite ratio. The infection was done in the presence or absence of activation stimuli (200 ng/ml LPS, 2 ng/ml IFN-γ) and the ROS production was measured with fluorometric assay using H_2_DCFDA probe. The values represent the mean ± SEM of three independents experiments. Indicated differences between groups are significant ^∗^*p* < 0.05, ^∗∗^*p* < 0.01, ^∗∗∗^*p* < 0.001.
