# Supplementary figures and images for: Dependency of B-1 Cells in the Maintenance of Splenic Interleukin-10 Producing Cells and Impairment of Macrophage Resistance in Visceral Leishmaniasis
Source: Front Microbiol. 2017 Jun 2;8:978. doi: 10.3389/fmicb.2017.00978 (PMC5454060; doi:10.3389/fmicb.2017.00978)

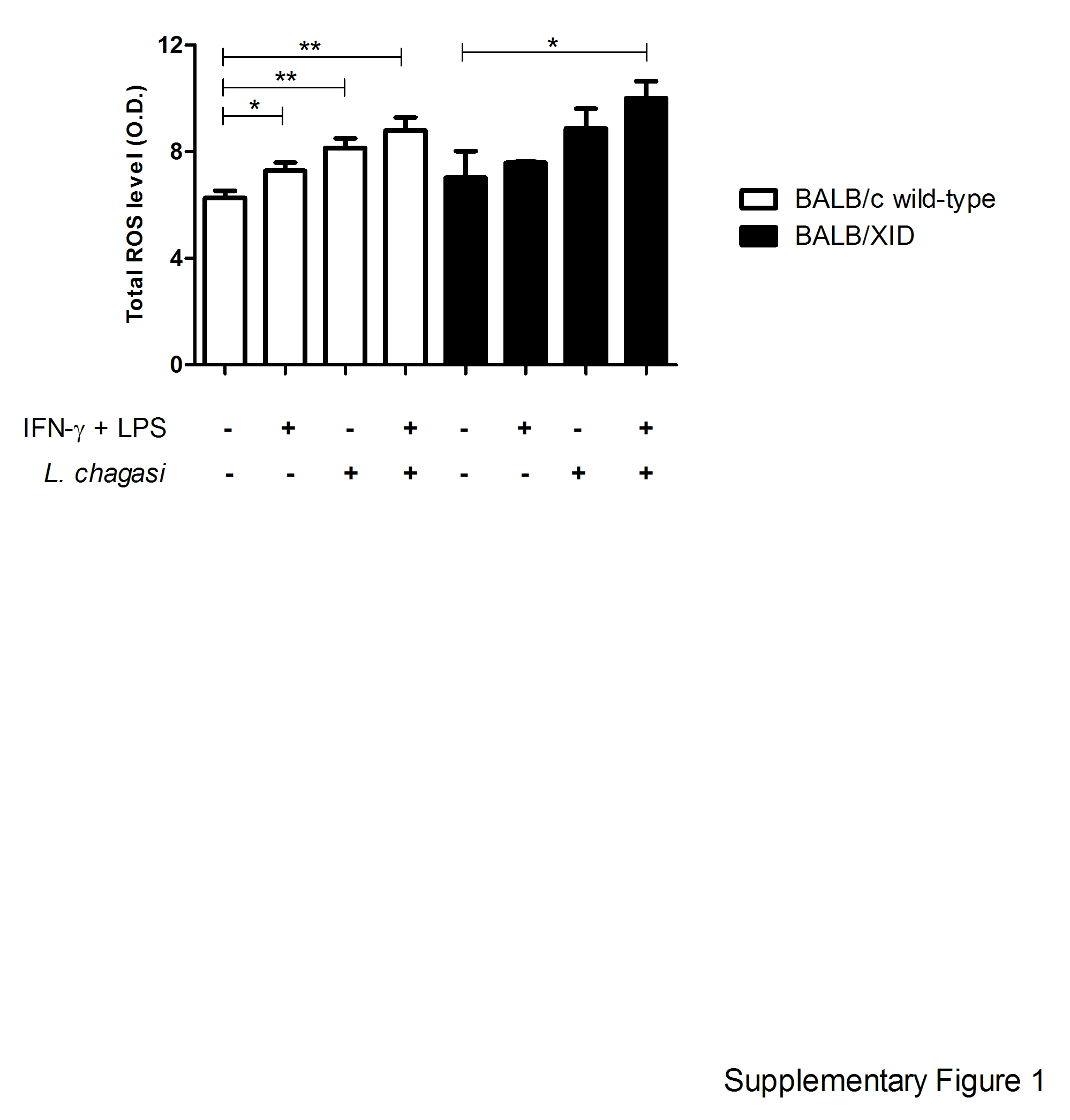

Supplement: FIGURE S1 — ROS production during Leishmania (L.) infantum chagasi promastigote infection. Resident peritoneal macrophages isolated from BALB/c or BALB/XID mice were infected with Leishmania (L.) infantum chagasi promastigote forms (at 1:10 host–parasite ratio. The infection was done in the presence or absence of activation stimuli (200 ng/ml LPS, 2 ng/ml IFN-γ) and the ROS production was measured with fluorometric assay using H2DCFDA probe. The values represent the mean ± SEM of three independents experiments. Indicated differences between groups are significant ∗p < 0.05, ∗∗p < 0.01, ∗∗∗p < 0.001. [file Image_1.TIF]
